# Archaea Carotenoids: Natural Pigments with Unexplored Innovative Potential

**DOI:** 10.3390/md20080524

**Published:** 2022-08-17

**Authors:** Antoine Grivard, Isabelle Goubet, Luiz Miranda de Souza Duarte Filho, Valérie Thiéry, Sylvie Chevalier, Raimundo Gonçalves de Oliveira-Junior, Noureddine El Aouad, Jackson Roberto Guedes da Silva Almeida, Przemysław Sitarek, Lucindo José Quintans-Junior, Raphaël Grougnet, Hélène Agogué, Laurent Picot

**Affiliations:** 1 UMR CNRS 7266 LIENSs, La Rochelle Université, 17042 La Rochelle, France; 2 UR 4312 CBSA, Université de Rouen Normandie, 27000 Évreux, France; 3 UMR CNRS 8038 CiTCoM, Faculté de Pharmacie, Université Paris Cité, 75006 Paris, France; 4 Research Team on Natural Products Chemistry and Smart Technologies (NPC-ST), Polydisciplinary Faculty of Larache, Abdelmalek Essaadi University, Tetouan, Larache 92000, Morocco; 5 NEPLAME, Universidade Federal do Vale do São Francisco, Petrolina 56304-205, Pernambuco, Brazil; 6 Department of Biology and Pharmaceutical Botany, Medical University of Lodz, 90-151 Łódź, Poland; 7 LANEF, Department of Physiology, Universidade Federal de Sergipe, São Cristóvão 49100-000, Sergipe, Brazil

**Keywords:** archaeal carotenoids, carotenoids, marine pigments

## Abstract

For more than 40 years, marine microorganisms have raised great interest because of their major ecological function and their numerous applications for biotechnology and pharmacology. Particularly, Archaea represent a resource of great potential for the identification of new metabolites because of their adaptation to extreme environmental conditions and their original metabolic pathways, allowing the synthesis of unique biomolecules. Studies on archaeal carotenoids are still relatively scarce and only a few works have focused on their industrial scale production and their biotechnological and pharmacological properties, while the societal demand for these bioactive pigments is growing. This article aims to provide a comprehensive review of the current knowledge on carotenoid metabolism in Archaea and the potential applications of these pigments in biotechnology and medicine. After reviewing the ecology and classification of these microorganisms, as well as their unique cellular and biochemical characteristics, this paper highlights the most recent data concerning carotenoid metabolism in Archaea, the biological properties of these pigments, and biotechnological considerations for their production at industrial scale.

## 1. Discovery of Archaea

**Figure 1:**
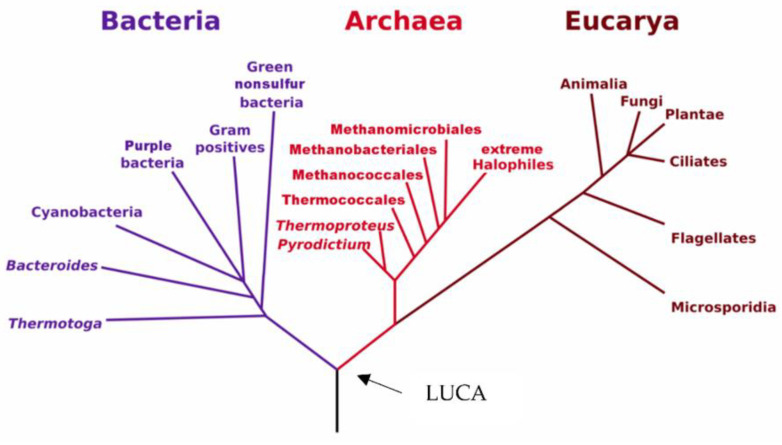
The phylogenetic tree of life, as redefined following the discovery of Archaea by Carl Woese. LUCA: Last Universal Common Ancestor [1].

In 1977, the American biologist Carl Woese had the groundbreaking idea of using the latest developments of molecular biology to sequence the ribosomal RNA of methane-producing anaerobic microorganisms, which were considered as extremophile bacteria. He discovered that these microorganisms were not bacteria, contrary to what their morphology suggested, but that they belonged to a third domain of life, totally distinct from bacteria and eukaryotes, which was named Archaea [1]. This pioneering discovery revolutionized the understanding of the living world, paved the way for the study and valorization of these extremophilic microorganisms and redefined with precision the phylogenetic tree of life (Figure 1). 

## 2. Ecology and Classification of Archaea

Archaea have mostly been isolated from extreme environments (oceanic hydrothermal vents, volcanic hot springs, salt lakes). However, it is now well documented that they also live in a wide range of common environments, including soil, seawater, marshes and human skin and gut. They play a major role in key ecological and geochemical processes, including the carbon cycle (especially methanogenesis), the nitrogen cycle (especially nitrification) and climate regulation. Their interaction with the human body is still poorly studied but may have physiological or pathophysiological importance, as they constitute an important part of the human microbiota present on skin and in the gastrointestinal tract. Archaea include extremely varied trophic types and can be divided into four major ecological groups, called (i) thermophiles and hyperthermophiles, (ii) halophiles, (iii) methanogens and (iv) psychrophiles.

(i) Thermophiles and hyperthermophiles live in hot, sulfurous springs such as geysers or underwater hydrothermal springs [2]. Thermophiles are defined by their ability to live at temperatures between 50 and 70 °C, while hyperthermophiles survive at temperatures above 70 °C and up to 113 °C. Among the hyperthermophiles, *Pyrodictium* is the genus of aquatic Archaea capable of surviving at the highest temperatures, with an optimal growth temperature ranging from 80 to 105 °C [2]. Because hot springs and sulfurous waters are mostly acidic, thermophiles and hyperthermophiles are also generally acidophilic and live at an optimal pH between 2 and 3, which involves physiological adaptations of membranes and cell walls to avoid acidic hydrolysis of their cellular constituents.

(ii) Halophilic Archaea have been isolated in hypersaline environments such as the Dead Sea and some inland lakes. They need a salinity of at least 1.5 M NaCl (87.66 g·L^−1^) to survive and can tolerate up to 5 M NaCl (292.2 g·L^−1^) or KCl (372.75 g·L^−1^). Some halophiles live in very alkaline environments, such as potash mines or lakes in which the pH is about 12, and are therefore called alkalophiles. Pigments produced by these halophilic organisms include phytoene, β-carotene, lycopene, haloxanthin, astaxanthin, canthaxanthin and bacterioruberin intermediates [3,4]. The main morphological types of halophiles are bacilli, cocci and irregular forms, but cells can also show several morphologies in the same culture and even square forms as observed for the species *Haloquadratum walsbyi* [5]. When the ionic conditions of the medium are altered, most cells lyse and the morphology of non-cocci cells can change depending on the salt concentration [6]. Halophilic Archaea are either mobile or immobile.

(iii) Methanogens are present in all environments, terrestrial or aquatic (swamps, rice fields, hot springs) as well as in the digestive tract of many animals. These Archaea are all strict anaerobes and produce methane by reduction of carbon dioxide. The genus *Methanopyrus* lives close to underwater hydrothermal springs, its optimal growth temperature is 98 °C, it dies from “cold” below 84 °C and can survive at 110 °C. *Methanopyrus* belongs to the phylum Euryarchaeota, order Methanopyrales. To date, it is the most thermophilic species known among the methanogenic Archaea.

(iv) Psychrophilic Archaea proliferate at 0–10 °C, metabolize in snow and ice at −20 °C, are predicted to metabolize at −40 °C and can survive at −45 °C [7,8]. Most of the Earth’s biosphere is cold (∼75%), and psychrophiles can be found in permanently cold environments (≤5 °C), such as alpine and polar habitats, the deep ocean, caves, permafrost, ocean subsurface and the upper atmosphere. In particular, psychrophiles are adapted to survive in the icy waters of the Arctic and Antarctic oceans. Psychrophiles are also present in seasonal and artificially cold (≤5 °C) environments, such as refrigerated appliances and products. They are also particularly abundant in the cold depths of the oceans (below 1,000 m) that cover nearly 70% of the planet’s surface.

## 3. Cell Biology and Biochemistry of Archaea

**Figure 2:**
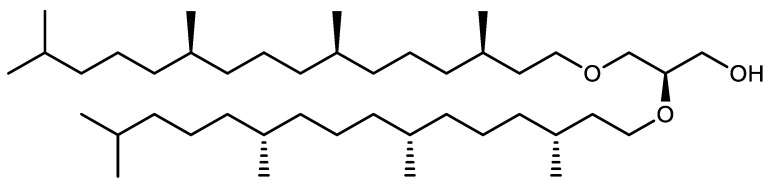
Structure of 2,3-di-*O*-phytanyl-sn-glycerol, a diether present in all Archaea membranes and considered characteristic of the reign [13].

Archaea are similar to bacteria in many characteristics, such as cell morphology, lack of nucleus, genomic organization and part of their organic metabolism. Depending on the species, their cell morphology can exhibit various shapes including squares, shells, rods, curved rods, filaments or various atypical shapes. Like bacteria, they divide by binary scission [9], contain a single circular chromosome and can contain one or more extra-chromosomal elements comparable to bacterial plasmids [10]. However, other aspects of their cell biology bring them closer to eukaryotes. In particular, mechanisms controlling archaeal DNA replication, transcription, translation and repair [11] are very similar to the machineries found in eukaryotes, and the proteins involved in these fundamental cell cycle events can be compared to their eukaryotic counterparts. However, previous reports on the biochemistry of Archaea have also revealed that these microorganisms exhibit unique biochemical and physiological features that make them remarkably different from bacteria and eukaryotes. One of the most important differences relies in their cell wall and membrane composition [12]. First, archaeal membranes do not contain sterols. Additionally, the fundamental and unique feature of archaeal membranes is that they contain original glycerol ethers with isoprenic and isopranic chains, especially phytanic acid, instead of classical glycerophospholipids esterified with fatty acids. These uncommon ethers can be divided into two main groups: diethers and tetraethers. In the first group, 2,3-di-*O*-phytanyl-sn-glycerol (Figure 2) is present in the membranes of all Archaea and therefore considered the “universal” archaeal lipid.

**Figure 3:**
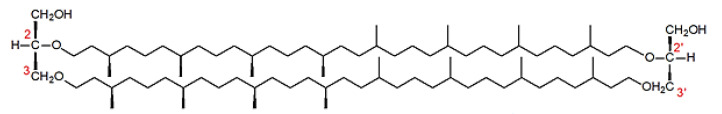
Structure of isocaldarchaeol, a tetraether present in the membrane of methanogens and thermophiles.

Glycerol tetraethers are particularly abundant in the membranes of methanogens and thermophiles. They are formed by two glycerol molecules linked by two C_40_ chains, themselves formed by head-to-head coupling of two phytane units. The fundamental characteristic of these glycerol ethers is that the central carbon of glycerol is etherified, leading to the formation of 2,3-sn ethers in Archaea (Figure 3) whereas 1,2-sn esters are present in the membranes of bacteria and eukaryotes.

The distribution of glycerol diethers and tetraethers has a chemotaxonomic significance for the classification of Archaea and supports the ecophysiological adaptation of cell membranes to extreme environmental conditions. Halophiles exclusively contain diethers while thermoacidophiles contain a very high proportion of tetraethers (percentages superior to or equal to 90). In methanogens, the percentages of diethers can vary from 40 to 100% [14]. Glycerol di- and tetraethers are associated with complex lipids that are also characteristic of the three first groups of Archaea according to the nature of their polar parts. Halophiles mainly contain phosphatidylglycerols, phosphatidylglycerophosphates and phosphatidylglycerosulfates. In contrast, the phospholipids of methanogens mainly consist in aminophospholipids and the complex lipids of thermophiles are essentially made of tetraethers. Glycerol ethers have various biological activities, and may be of particular interest as anticancer natural products [15].

## 4. Archaea Pigments: Bacteriorhodopsin and Carotenoids

Archaea also represent a valuable source of novel pigments, including carotenoids (detailed further below) and proteins called bacteriorhodopsins.

### 4.1. Bacteriorhodopsin

Some halophilic Archaea integrate a purple pigment, made of an assembly of opsin with retinal, an apocarotenoid. This complex, denominated as bacteriorhodopsin (BR), crosses the cell membrane and is strongly bound to it through hydrophobic interactions. BR is a seven-α-helical protein containing a chromophore molecule (retinal). It shares structural similarities to the mammalian GPCR protein rhodopsin and is a light-dependent proton pump. Light photons activate the pump to produce ATP by creating a proton gradient across the membrane [16,17]. The word *archaerhodopsins* is also used to designate this family of proteins and to specify that they are synthesized by Archaea.

*Halobacterium salinarum* bacteriorhodopsin was the first microbial rhodopsin documented [18] and it has been broadly studied as a model for membrane protein structure and activity [19,20,21]. *Halobacterium salinarum* is the most studied Archaea for the production of bacteriorhodopsin [22]. This halophilic species grows in high salinity environments such as salt crystallization ponds and often reaches densities of 10^7^ to 10^8^ cells·mL^−1^ [23]. At this high density, oxygen availability is reduced and aerobic metabolism is almost impossible. In this sense, the synthesis of retinal and bacteriorhodopsin allows the species to be autonomous for its ATP production through photosynthesis.

The biotechnological interest of bacteriorhodopsin lies in its ability to exist in two stable forms at two different wavelengths, allowing its use to be to considered in “biological computer” projects. Bacteriorhodopsin could indeed be used as an extremely miniaturized memory storage unit controlled by light pulses (at a rate of one bit per molecule, a 12 cm-diameter disk could hold 20 to 50 TB). The use of bacteriorhodopsin is one of the first applications of organic molecular electronics, an emerging discipline in nanocomputing [24].

Bacteriorhodopsin is also a likely candidate for integration into nanostructures. It is a light collector that can effectively be used as a solar collector to collect light energy, that could either be consumed directly or stored in batteries. The ability of bacteriorhodopsin to convert light energy into chemical energy or sunlight into electricity has been used in various applications, including optical appliances but also therapeutic/medical applications and research [25,26,27,28].

Various therapeutic uses of this protein have been considered, including the treatment of degenerated retinal blindness and eye disorders, use as adjuvant in vaccination protocols, treatment of malignant tumors, regulation of gene transcription, modulation of drug delivery, transport and release of drugs, cell signaling, apoptosis of neoplastic cells, cell signaling disruption, manufacture of neuro-stimulation devices and pharmaceutical applications [17,29,30,31,32].

### 4.2. Archaea Carotenoids

#### 4.2.1. Overview of the Structure and Function of Carotenoids

Carotenoids are natural pigments that have received particular attention because of their ecophysiological function, biotechnological applications, and their potential beneficial effects on human health [33,34,35,36,37,38,39,40,41,42,43,44]. The color of these molecules can range from colorless to red, through various yellow-orange tones, and they represent the second most abundant natural pigments in nature [45,46]. For example, fucoxanthin, an epoxycarotenoid found in brown microalgae and seaweeds, is the major carotenoid present in marine ecosystems, representing 10% of the total carotenoid production [34]. Apocarotenoids, such as retinal, are defined as derivatives of carotenoids produced by chemical or enzymatic oxidative cleavage.

The most important ecophysiological function of carotenoids is photosynthesis, as they actively participate in the harvesting of photons in the light-harvesting complexes of photosynthetic organisms. They are therefore found in all phototrophic bacteria, cyanobacteria, algae and land plants. Their biological activities have been studied in detail in photosynthetic organisms and include other important ecophysiological functions such as membrane stabilization, photoprotection and antioxidant activities. Animals, including humans, consume phototrophic organisms and utilize carotenoids or apocarotenoids for important physiological functions, including body coloration, sexual attractiveness, vitamin A production, antioxidant activity, transcription factor activation and photoreception. Pharmacological studies have also demonstrated the diverse biological activities of carotenoids and apocarotenoids, including antimicrobial, antifungal, antidiabetic, immunostimulant, anti-obesity, anti-inflammatory, anticarcinogenic and cancer-preventive, antimetastatic, antiangiogenic, radioprotective, anti-atherosclerotic, neuroprotective and chemosensitizing properties of multidrug-resistant cancer cells [37,39,41,43,44,47,48,49,50,51,52,53,54]

Due to their color and health-promoting properties, carotenoids find a wide range of applications in the food, pharmaceutical, nutraceutical and cosmeceutical industries [55].

**Figure 4:**
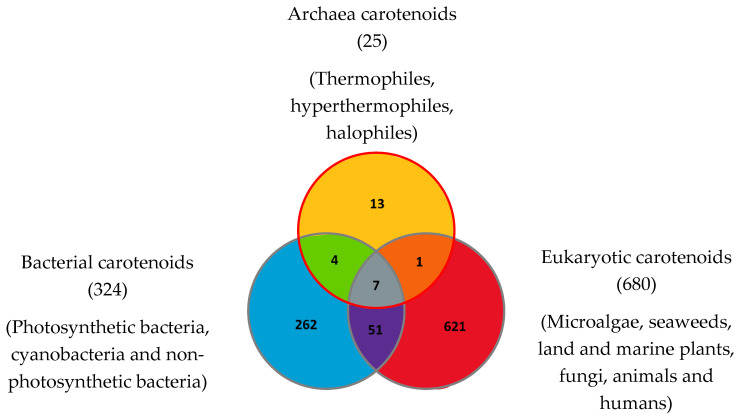
Carotenoids and apocarotenoids in the three domains of life. A total of 1204 carotenoids and apocarotenoids have been identified, some of them being exclusive to one of the three domains of life and some of them being shared [47]. For 245 carotenoids and apocarotenoids, the Japanese carotenoids database gives no indication of the producer organisms [47].

To date in 2022, 1204 natural carotenoids and apocarotenoids have been described in the three domains of life [56]. These include 324 carotenoids present in bacteria (from which 262 were exclusively found in bacteria), 680 carotenoids in eukaryotes (from which 621 are exclusive to eukaryotes) and 25 carotenoids in Archaea (including 13 exclusively found in Archaea) (Figure 4). Bacterial carotenoids and apocarotenoids are synthesized by photosynthetic bacteria, cyanobacteria and non-photosynthetic bacteria. All or most Archaea synthesize carotenoids. Eukaryotic carotenoids and apocarotenoids are present in photosynthetic carotenogenic species (microalgae, seaweeds, land and marine plants), non-photosynthetic carotenogenic species (fungi) and non-photosynthetic non-carotenogenic species (animals and humans) that obtain them by the consumption of producing species. Given this biodiversity, the number of species containing and/or synthesizing carotenoids and apocarotenoids is immense and impossible to determine exactly.

**Figure 5:**
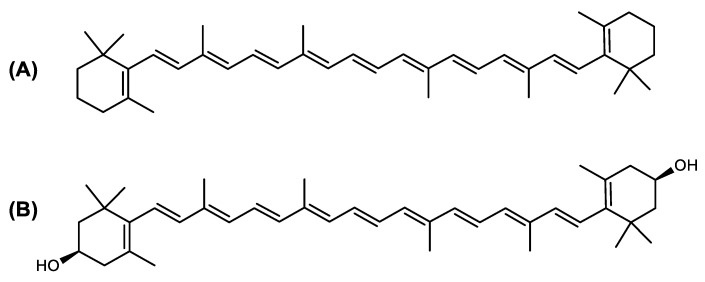
2D structures of model carotenoids. (**A**) β,β-carotene (CID: 5280489) and (**B**) zeaxanthin (CID: 5280899). Chemical 2D were obtained from PubChem (NIH).

The carotenoid family mostly consist of C_40_ hydrocarbon backbone molecules (1121 molecules) but also includes a smaller number of molecules containing 30 (37 carotenoids), 35 (5 carotenoids), 45 (13 carotenoids) and 50 carbon atoms (33 molecules). The C_40_ carotenoids are formed by the polymerization of 8 isoprene units, which are often modified by various oxygen-containing functional groups to obtain cyclic or acyclic xanthophylls. Thus, their polarity can vary from highly hydrophobic to amphiphilic or relatively polar. All carotenoids possess a long chain of conjugated double bonds and a near bilateral symmetry around the central double bond, as common chemical features [57]. According to the absence or presence of oxygen atoms, carotenoids are basically classified into carotenes or carotenoid hydrocarbons, composed of carbon and hydrogen only (e.g., lycopene and β,β-carotene) and xanthophylls or oxygenated carotenoids that can contain epoxy, carbonyl, hydroxyl, methoxy or carboxylic acid functional groups (e.g., lutein, canthaxanthin, zeaxanthin, violaxanthin, capsorubin, fucoxanthin and astaxanthin) [58,59]. Some xanthophylls exist as fatty acid esters, glycosides, sulfates, and can be found associated to protein complexes such as rhodopsin. Figure 5 presents the 2D structures of two model carotenoids, one carotene and one xanthophyll.

The absorption properties of each carotenoid are determined by the level of conjugation and the isomerization state of the backbone polyene chromophore. Carotenoids occur as diverse stereoisomers with various chemical and physical properties, due to the numerous conjugated double bonds and cyclic end groups. The most important are geometric isomers (*E-/Z-* or *trans-/cis*-). A double bond links the two residual parts of the molecule either in an *E*-configuration with both parts on opposite sides of the plane, or a *Z*-configuration with both parts on the same side of the plane. UV-Vis spectrophotometry, NMR or Raman spectroscopy can be used to differentiate the carotenoids’ isomers [60]. Although some *Z-(cis)* isomers of carotenoids could be isolated from marine microorganisms, including Archaea, it is important to note that these molecules might be produced by photoisomerization during the extraction, which does not allow us to affirm their presence in the living cell before extraction.

In microorganisms, carotenoids play a key role in the processes of photoprotection, photosynthesis, phototropism and resistance to oxidative stress. Some carotenoids have been described as virulence factors in bacteria, permitting the cells to counter the oxidative stress caused by phagocytes (e.g., staphyloxanthin produced in *Staphylococcus aureus*). Importantly, some carotenoids, such as salinixanthin or thermozeaxanthin, are only produced by extremophilic microorganisms [61,62,63], suggesting a potential function in thermal, pH or salinity adaptation. Carotenoids are widely known for their remarkable antioxidant properties [64] which are attributed to their double bond structure and ability to delocalize unpaired electrons [65]. Therefore, carotenoids are able to quench free radicals, such as superoxide (O_2_•^−^), hydroxyl (OH•) and peroxyl (ROO•) radicals [66]. Reactive nitrogen species (RNS) and reactive oxygen species (ROS) are metabolic by-products generated by almost all biological systems. When there is an imbalance in favor of ROS or RNS production, most biomolecules and cellular structures are negatively affected by excessive oxidation or nitration of lipids, nucleic acids and proteins. Over the years, it has been much detailed that oxidative stress is one of the reasons for the beginning and progression of many diseases, including cancer, heart disease or diabetes. From this perspective, the high antioxidant activity of dietary carotenoids likely explains their cancer-preventive effect, as they can protect cellular macromolecules from oxidation and thus limit inflammation [67]. Carotenoids also modulate gene expression and have anti-inflammatory and immunomodulatory activities [68] that limit the emergence of cancer cells. Additionally, extremophilic microorganisms that live in solar salt flats (halophilic microorganisms) are exposed to high amounts of oxidative stress due to intense solar radiation or high temperatures (up to 50 °C in summer). In response to this stress, they have evolved the synthesis of carotenoids, which are very active in neutralizing ROS [69].

A number of in vivo studies have also demonstrated that carotenoids slow down tumor growth and metastasis and have antiangiogenic activity. The ability of carotenoids to prevent cancer or limit its spread needs to be examined in relation to their behavior in various cellular and tissue environments. In normal tissues, where inflammation and ROS concentration are very low, carotenoids prevent cancer because of their antioxidant activity. However, this ability to neutralize ROS has a downside, as carotenoids oxidize themselves instead of biological macromolecules, leading to the production of oxidized derivatives such as carotenals and apocarotenals. This is especially the case in high inflammatory environments. For example, β-carotene supplementation in smokers increases the risk of lung cancer, as pro-carcinogenic oxidized apocarotenal derivatives are produced by chemical reaction with the pro-oxidative polyaromatic hydrocarbons contained in cigarette smoke [70]. Carotenoid supplementation for the prevention or treatment of cancer should thus be considered with caution, depending on the inflammatory and pro-oxidative status of tissues. The contribution of pro-oxidative metabolites to the initiation or prevention of cancer is still a matter of discussion among researchers. Recent studies have provided evidence on the pro-oxidative activity of some carotenoids (especially epoxycarotenoids), which could partly explain their cytotoxic and pro-apoptotic activity in cancer cells [71]. The pro-apoptotic activity of carotenoids in cancer cells may be associated with their ability to incorporate the cell membrane, sensitize lipids to oxidation, alter membrane fluidity, and interfere with cell signaling pathways. Tumor cells can have different redox statuses, from highly sensitive to very resistant to ROS, which may also partly explain the variable cytotoxicity of carotenoids depending on the cell line considered. Carotenoids have been shown to promote the PI3K/Akt and nuclear factor erythroid 2 (Nrf2) signaling pathways [72], inhibit NF-kB, p38 MAPK, and JAK-2/STAT-3 signaling pathways, which are also linked to inflammation and tumorigenesis. Furthermore, carotenoids can increase gap junction formation, a cellular process that may contribute to their anti-carcinogenic properties [73]. At last, an anti-adiposity activity has also been documented for some carotenoids such as cantaxanthin or fucoxanthin, through the stimulation of lipolysis in adipocytes [74].

Given their many attractive biological and pharmacological activities, societal demand for carotenoids has increased dramatically in recent years, especially for the food, pharma and cosmetics markets. In this view, the biosynthesis and purification of carotenoids from large-scale cultures of microorganisms are broadly studied, and a specific interest exists for the production of novel molecules produced by bacteria, marine living organisms and Archaea.

#### 4.2.2. Ecophysiological Function of Carotenoids in Archaea

Archaea, especially Haloarchaea, mostly synthesize an uncommon C_50_ carotenoid called bacterioruberin (BR) and its intermediates: bisanhydrobacterioruberin (BABR), monoanhydrobacterioruberin (MABR), and 2-isopentenyl-3,4-dehydrorhodopin (IDR) [75,76,77,78]. These 50-carbon carotenoids function to increase membrane rigidity [79] and provide protection against UV light [80]. The presence of carotenoids in the membrane of archaeal cells might assist cells to adapt to hypersaline conditions by acting as a water barrier, allowing ions and oxygen molecules to pass through the cell membrane. Therefore, these carotenoids can stabilize archaeal cells under high osmotic and oxidative stresses [81,82]. Despite the fact that β-carotene, lycopene, and phytoene have been identified in haloarchaeal extracts, they are present at low concentrations [76,83]. Those carotenoids are located in the cell membrane and are responsible for the color shown by the red colonies when haloarchaea cells develop on solid media or the red color that can be observed in the close environment of salted lakes such as Torrevieja lake in Spain, for example. Carotenoids also contribute to the modulation of the physicochemical properties of membranes [84]. For example, bacterioruberin is a fundamental component of specific transmembrane proteins [85] and controls membrane organization through the modulation of membrane dynamics and physics [79]. It is also assumed that carotenoids exert antioxidant activities and regulate membrane functions in Archaea, especially in extremophile species.

#### 4.2.3. Focus on Archaea Carotenoids Structures, Biosynthesis Pathways and Archaea Producing Species

##### Structure of Archaea Carotenoids

Archaea produce 25 different carotenoids and apocarotenoids, 13 of which are specific to this domain of life [56]. In total, 4 of the 25 molecules are produced by both bacterial and archaeal domains and only one carotenoid is produced by both eukaryotes and Archaea. Table 1 lists the 25 carotenoids synthesized by Archaea and highlights the ones shared with eukaryotes, bacteria, or both. Table 2 and Table 3 present the chemical structure of Archaea carotenoids, producing species and the carotenoid composition in the various Archaea ecophysiological groups.

##### Biosynthesis of Carotenoids in Archaea

All carotenoids are derived from isopentenyl pyrophosphate (IPP) and its isomer, dimethylallyl pyrophosphate (DMAPP) [88]. The biosynthetic pathway from isopentenyl pyrophosphate (IPP) to lycopene is common to prokaryotes. The first reaction performed by a farnesyl diphosphate synthase FDPS (2.5.1.10) converts IPP to farnesyl pyrophosphate (FPP). Next, the enzyme CrtE (2.5.1.29), a geranylgeranyl diphosphate synthase, synthesizes geranylgeranyl diphosphate (GGPP). From this molecule, phytoene is obtained by the action of a 15-cis-phytoene synthase called CrtB (2.5.1.32). Lycopene is generated from phytoene by a series of desaturations catalyzed by phytoene desaturase CrtI. From lycopene, some Archaea have specific enzymes and pathways that lead to the synthesis of Archaea-specific carotenoids. These enzymes are described in the Kyoto Encyclopedia of Genes and Genomes (KEGG) and identified by their identification number in parenthesis in the metabolic pathways [89].

##### Carotenoids Biosynthesis in *Haloarcula japonica*

**Figure 6:**
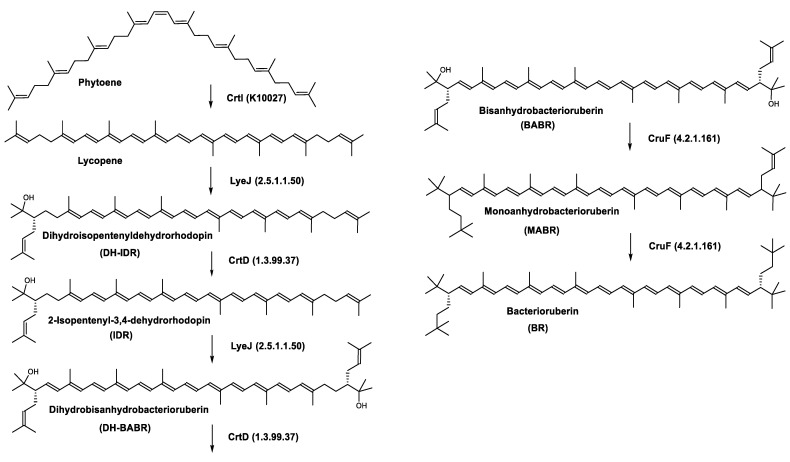
Carotenoids biosynthesis pathway in *Haloarcula japonica* [87]. Numbers in parenthesis indicate the enzyme number or EC number.

*Haloarcula japonica* is a predominantly triangular, disc-shaped, extremely halophilic Archaea that requires high concentrations of NaCl for growth [97]. In this species, lycopene is generated from phytoene via a series of desaturation reactions that are catalyzed by phytoene desaturase (CrtI) (Figure 6). Subsequently, a bifunctional lycopene elongase and 1,2-hydratase (LyeJ) perform the next reactions, resulting in dihydroisopentenyldehydrorhodopin (DH-IDR). In the next step, CrtD forms double bonds at C-3,4 of the lycopene derivative which leads to the 2-isopentenyl-3,4-dehydrorhodopin (IDR). CrtD was characterized as a carotenoid 3,4-desaturase. The next two steps are the same as the previous ones and result in the formation of dihydrobisanhydrobacterioruberin (DH-BABR) with the LyeJ enzyme and then bisanhydrobacterioruberin (BABR) through the activity of the CrtD enzyme.

The last two steps are catalyzed by a C_50_ carotenoid 2″,3″-hydratase (CruF) which generates bacterioruberin (BR). It has been suggested that the antioxidant capacity of carotenoids is linked to the number of conjugated double bonds and hydroxyl groups [98,99]. In this view, bacterioruberin, which contains 13 conjugated double bonds and 4 hydroxyl groups, is a highly effective free-radical scavenger.

**Figure 7:**
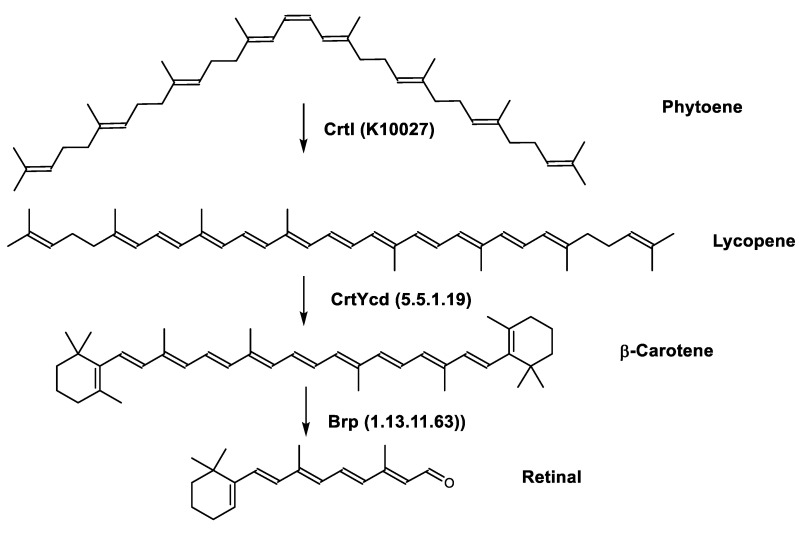
Biosynthetic pathways of retinal (apocarotenoid) in *Haloarcula japonica* [87]. Numbers in parenthesis indicate the enzyme number or EC number.

Retinal is also synthesized by *Haloarcula japonica*. In the same method as in the biosynthetic pathway of bacterioruberin, lycopene is produced from phytoene through a series of desaturation reactions catalyzed by phytoene desaturase (CrtI). Retinal is synthesized through the cyclization of lycopene to β-carotene by lycopene β-cyclase (CrtYcd) [100] and the subsequent cleavage of β-carotene to retinal by β-carotene dioxygenase (Brp) [101] (Figure 7).

##### Regulation of the Bacterioruberin Synthesis

Lycopene is the last common intermediate for retinal and bacterioruberin biosynthesis [102]. CrtYcd, the lycopene cyclase, catalyzes the conversion of lycopene to β-carotene that is subsequently cleaved to form retinal. LyeJ, the lycopene elongase catalyzes the prenylation of lycopene to the first bacterioruberin intermediate. Therefore, lycopene is a key intermediate that may be used either for retinal or bacterioruberin biosynthesis and the LyeJ enzyme is a potential target for regulation of these pathways.

**Figure 8:**
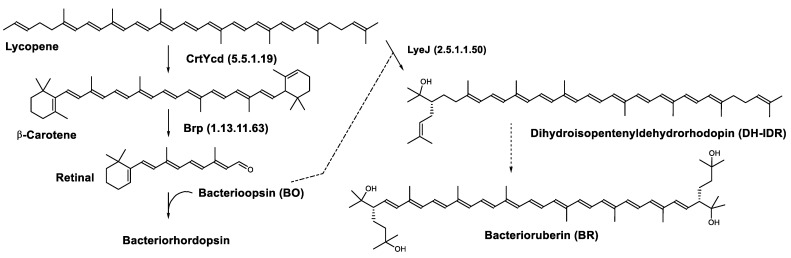
Biosynthesis of bacteriorhodopsin and bacterioruberin in *Halobacterium salinarum* [102]. It is hypothesized that bacterioruberin synthesis is inhibited by bacterioopsin as indicated by the dashed line. Numbers in parenthesis indicate the enzyme number or EC number.

In the haloarchaea *H. salinarum*, when bacterioopsin (BO) is not linked to the retinal, LyeJ activity is repressed, so that bacterioruberin production is reduced and lycopene is available for the synthesis of retinal. The regulation of retinal and BO synthesis is coordinated in Archaea (*H. salinarum* for example) through the control of gene transcription. Low oxygen pressure results in the induction of the *bop* gene, which encodes bacterioopsin (BO) [103,104]. This increased transcription of the *bop* gene is mediated by the bacterioopsin gene activator (*Bat*), a transcription factor that also activates the transcription of genes encoding the retinal biosynthetic enzymes [105]. A study suggested that another transcription factor, *brz*, is also required for the induction of BO and related enzymes [106]. The most plausible explanation for these findings is that BO, in the absence of its retinal cofactor, directly binds LyeJ, and that when sufficient retinal is available to convert BO to bacteriorhodopsin, LyeJ is released and catalyzes the conversion of lycopene to bacterioruberin [102] (Figure 8).

This regulatory mechanism plays a major role in the response of *H. salinarum* and possibly haloarchaea in general to environmental changes. Opsin-based inhibition of bacterioruberin biosynthesis is a widely distributed mechanism in Archaea and Bacteria. In addition, interactions between opsins and other proteins are numerous and raise the possibility that this regulatory mechanism confers a selective advantage on organisms that express ion-pumping rhodopsins.

##### Carotenoids Biosynthesis in *Sulfolobus shibatae*

**Figure 9:**
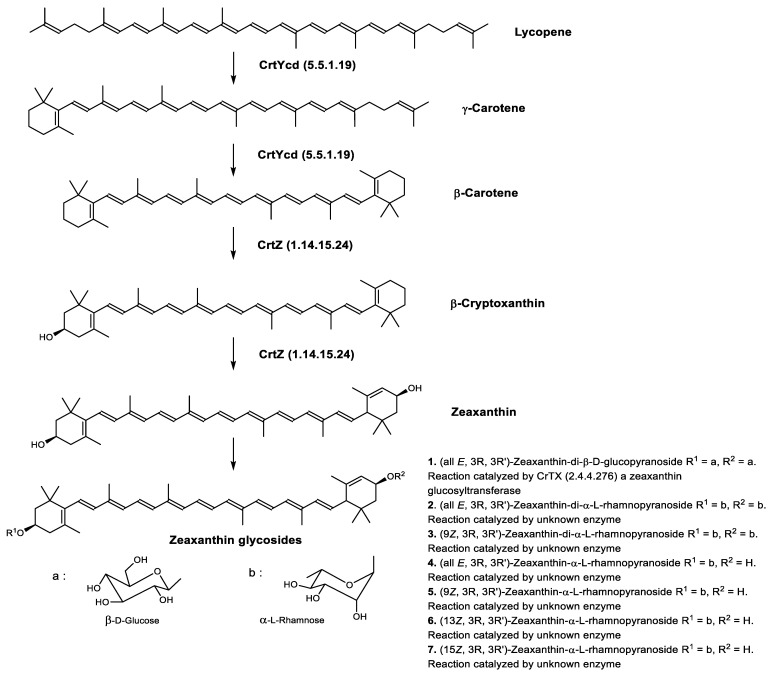
*Sulfolobus shibatae* carotenoids reconstructed pathways [90]. The bold numbers 1. to 7. correspond to all identified zeaxanthin glycosides. Numbers in parenthesis indicate the enzyme number or EC number.

Zeaxanthin glycosides are the major carotenoids present in *Sulfolobus shibatae*. The (all-*E*)-isomers 1, 2, and 4 (Figure 9) had been found before in bacteria, algae, and other organism, but never in an archaeal species [107]. Naturally occurring (*Z*)-isomers of zeaxanthin glycosides were first reported in 1989. It can be speculated that these glycosides act in *Sulfolobus* as membrane reinforcers, as previously proposed for bacteria [107]. Although shorter than C_50_-carotenoids, their short length may be compensated by the presence of the carbohydrate moieties to act as membrane reinforces.

Table 4 provides a summary of the enzymes and relative genes involved in carotenoid biosynthesis in Archaea.

#### 4.2.4. Biological Activities of Archaea Carotenoids

Table 5 summarizes the biological activities described in the literature for carotenoids and apocarotenoids produced by Archaea.

#### 4.2.5. Biotechnological Considerations for the Production of Archaea Carotenoids

Because of the extreme growth conditions of the majority of Archaea species, the development of laboratory culture conditions has been a challenge, and only a small amount of studies discussing the growth and production of Archaea have been published to date. Archaea have historically been eclipsed by bacteria and eukaryotes in terms of public attention, industrial uses and scientific studies, although their biochemical and physiological characteristics offer possibilities for a wide range of biotechnological applications, including the production of polysaccharides, biomaterials, thermophilic enzymes, and novel pigments [136].

Carotenoids represent a significant fraction of lipids, such as 0.2% (*w*/*w*) in *Haloferax alexandrinus*, an extreme halophile, most of them being bacterioruberin and canthaxanthin with small quantities of 3-hydroxyechinenone and β-carotene [137]. Likewise, important quantities of bacterioruberin were found in another extremely halophilic Archaea, *Haloarcula japonica* [45]. In contrast, only zeaxanthin was identified in *Sulfolobus shibatae,* a thermoacidophilic Archaea [90].

A number of archaeal enzymes have also received attention from researchers due to their unique characteristics and potential for research and industrial applications. This is the case for thermostable thermophilic and hyperthermophilic enzymes (extremozymes) such as DNA polymerases that are used for polymerase chain reaction (PCR) and are not damaged by the high temperatures used to separate the DNA strands during the DNA amplification phase. The three most commonly used DNA polymerases come from the species *Pyrococcus furiosus* (Pfu polymerase), *Thermococcus littoralis* (Tli or Vent polymerase) and *Thermus aquaticus* (Taq polymerase) [138,139]. Archaea appear also as an interesting source of original bioactive molecules, such as polysulfur heterocycles, that could find interesting pharmacological applications [15].

From a biotechnological perspective, the extreme conditions in which Archaea grow (pH, salt concentration and high temperature) reduce the risk of culture contamination by other microorganisms and allow their cultivation under non-sterile conditions, thus reducing cultivation costs. Some Archaea can tolerate the use of toxic solvents and metabolites, which are known to be lethal to potential contaminants. It can be assumed that considering the small number of studies dealing with the isolation of bioactive molecules from Archaea, many molecules of biotechnological or pharmacological interest still remain to be identified.

One of the advantages of haloarchaea for C_50_ carotenoid production is that their biosynthesis can be easily enhanced by switching the cells from a high-salt culture medium that favors growth (20–25% *w*/*v*) to a lower-salt culture medium (less than 16% *w*/*v*) that promotes rapid accumulation of bacterioruberin [77,140,141]. Therefore, feasible carotenoid production from haloarchaea should preferably be carried out by a two-step process consisting of biomass production under high salt concentrations (first step) followed by rapid carotenoid biosynthesis and increased accumulation under low salt concentrations (second step).

One of the main challenges faced by chemical and mechanical engineers in universities and biotechnology companies when developing new fermentation equipment is the need to mimic, in the laboratory, the extreme environments in which extremophiles have been collected. The materials must withstand high temperatures as well as low pH for the cultivation of thermoacidophiles such as *Sulfolobus solfataricus*, but also high salinity or highly alkaline solutions for halophiles such as *Haloarcula japonica* and alkalophiles.

Although the high concentration of salt in the medium corrodes stainless steel fermenters and piping systems, inexpensive materials such as plastic, carbon steel and ceramics can be used to build fermenters and piping systems to avoid corrosion, since high-pressure sterilisation is not required. Once the pigments have accumulated inside the haloArchaea cells, the next step to complete the production process is the extraction from the biomass. When carotenoid production is carried out in microalgae cells, which are well-known natural producers of carotenoids, extraction can become a key limitation in terms of process costs. The cells of many microalgae species are difficult to break down due to their cell wall composition which is highly resistant to standard cell-breaking tools, including the freezing and thawing of algal pellets in liquid nitrogen or the use of sonication. One of the main advantages of haloarchaeal species for carotenoid extraction is that low concentrations of salt cause cell lysis, thus avoiding the energy investment required to enable efficient cell disruption [137] and making carotenoids readily available for solvent extraction compared to direct extraction from unbroken cells. This means that haloarchaeal cells may be suitable for maximizing pigment recovery at lower costs compared to other microorganisms.

The massic yield of carotenoids in haloarchaea is mainly dependent on the strain and culture conditions used. The total carotenoid content in *Haloarcula japonica* was 335 μg·g^−1^ dry mass [45], and the contents in *Halobacterium salinarum* and *Halococcus morrhuae* were 89 and 45 μg·g^−1^, respectively [61]. A major limitation to the industrial application of Archaea enzymes and metabolites is the low productivity of the fermentation processes as a result of low growth rates and low biomass production yields. To overcome these limitations, attention has been focused on studying the physiology of Archaea of biotechnological interest and on designing bioreactors and bioprocesses that increase productivity.

In physiological studies, continuous cultures have often been reported as effective systems, especially for demonstrating the correlation between substrates and biosynthesis of enzymes and other metabolites. In fact, the non-conventional environmental conditions necessary for the culture of Archaea allow long-term experiments without contamination problems. Optimization of the growth medium is also of key importance in the production of extremophilic biomasses for subsequent exploitation in industry. Continuous culture experiments are fundamental in clarifying the metabolism of particular substrates and the significance of specific enzymes in unconventional biochemical pathways. This operational model has also helped in delineating the key growth requirements for a variety of extremophilic microorganisms, which often were demonstrated to require special minerals, amino acids or vitamins to grow at reasonable rates.

For the production of extremozymes, gene cloning in a mesophilic host easy to cultivate [142] is of crucial interest, especially for the simplification of downstream processing. Indeed, specific procedures, based on the difference in stability under extreme conditions, can be easily developed to purify the host protein product. Genes coding for several extremozymes have been cloned into heterologous hosts, with the aim of overproducing the enzyme. The best results could be obtained by applying recent molecular biology techniques and new fermentation strategies. Concerning lipids, archaeal molecules are unique in term of structure and relation to their topology and function in membranes. This singularity is of interest for the biotechnological applications of these compounds as they can be used for the formation of liposomes with remarkable thermostability and tightness against solute leakage [138]. In vitro and in vivo studies have shown that archaeosomes (liposomes from archaeal lipids) are safe and that their stability offers superior alternatives for several biotechnological applications, including drug delivery systems, gene delivery systems or cancer imaging agents [143]. Halophilic microorganisms, in particular *Halomonas* spp., are also interesting for the production of various products used in different industrial domains. Many halophiles are capable of accumulating polyhydroxyalkanoates (PHAs) [144], a family of biodegradable and biocompatible polyesters that are developed in an industrial value chain ranging from bioplastics, biofuels, fine chemicals to medicine [145,146]. PHA accumulation by halophiles was first documented in 1972 [147] and other halophiles were further exploited and found to synthesize high amounts of PHAs [148,149,150]. The use of halophiles for PHA production can reduce the costs of fermentation and recovery processes because of the high salt concentration that prevents contamination by non-halophiles, and the limits energy consumption and complexity of the sterilization process. In addition, the cell membranes of haloarchaea are easy to lyse in the absence of salt, especially when using distilled water. This osmotic shock makes it much easier and cheaper to recover PHAs from crude extracts. In this view, halophiles provide an inexpensive platform for the biotechnological production of PHAs at industrial scale.

### 4.3. Focus on Bacterioruberin Biological Activities

The ecophysiological function of bacterioruberin is to serve as a photon sensor in Archaerhodopsin-2 (aR2), a retinal protein–carotenoid complex present in the membrane of *Halorubrum* sp. This complex functions as a light-driven proton pump and ensures ATP production for the cell [85,151,152,153]. In addition, it was shown that *Haloferax mediterranei* (Order: Halobacteriales) is able to counteract oxidative stress generated by high concentrations of hydrogen peroxide through the production of bacterioruberin. Bacterioruberin successfully neutralizes hydrogen peroxide, confirming that cells use this carotenoid to maintain oxidative balance and that this compound is indeed very effective against ROS [83]. Studies with *Halobacterium halobium* carotenoid extracts have also demonstrated the antiproliferative activity of bacterioruberin in human cancer cell lines HepG2 (hepatocarcinoma) and MCF-7 (breast) [154], associated to the activation of caspases and induction of apoptosis. Half of current anticancer drugs are derived from natural compounds or their mimetics [155] and carotenoids are of great interest due to their lack of oral toxicity. A dose-dependent antiproliferative effect in HepG2 cells has also been reported for carotenoid extracts from *Haloplanus vescus* (62.5 nM–1 μM) and *Halogeometricum limi* (approximately 1 μM). An anti-haemolytic activity of bacterioruberin was also demonstrated in vitro [156] and bacterioruberin is a potent inhibitor of matrix metalloproteinase 9 (MMP-9) [157]. MMP-9 is one of the key proteases involved in cancer metastasis and angiogenesis [158]. The mechanisms involved in the anticancer activity of bacterioruberin are currently unclear and the fact that the same molecule can have both antioxidant and pro-oxidant activity has been debated within the scientific community. Therefore, its value in treating tumors in humans remains to be clearly established [159]. To date, no studies have been performed to evaluate whether bacterioruberin is resorbed in animal or human models and no information exists regarding its toxicity. However, given its photoprotective and antioxidant properties, the possibility of using it for cosmetic applications and in particular for sunscreen products seems realistic.

Finally, Zalazar et al. tested whether bacterioruberin extracts from a genetically modified *Haloferax volcanii* (Order: Halobacteriales) could reverse the damage caused by the freezing and thawing of ram sperm. Bacterioruberin extracts exerted beneficial effects on sperm viability by reducing the apoptotic and necrotic population, as well as improving sperm motility, presumably by stabilising cell membranes after thawing [160].

## 5. Conclusions

In this review, we have highlighted the potential of Archaea for the synthesis of novel carotenoids that could find various biotechnological and pharmaceutical applications. Archaea are interesting microorganisms for the production of carotenoids on a large and medium scale, as fast and cheap culture systems can be coupled with easy downstream extraction and purification processes. The extreme culture conditions limit the risk of contamination by other micro-organisms. The engineering of haloarchaea is now possible thanks to the knowledge of the carotenogenesis metabolic pathways, sequencing of the genomes of haloarchaea and the possibility of genetic manipulation of haloarchaea. Carotenoid production by haloarchaea can also be improved by optimizing the culture medium in terms of salinity, pH and temperature. Carotenoids produced by halophilic Archaea can play the dual role of membrane stabilizers and highly antioxidant compounds, making them essential compounds for the survival of these microorganisms but also represent molecules with significant potential for health, cosmetic and biotechnological applications. The small number of studies dedicated to the biosynthesis of natural products from Archaea suggests that this field of research is still relatively unexplored and that numerous discoveries will be made in the years to come.

## Figures and Tables

**Table 1:** Carotenoids and apocarotenoids synthesized by Archaea: A total of 25 molecules were isolated from Archaea, including 1 shared with eukaryotes, 4 shared with bacteria and 6 common to the 3 domains of life. In total, 13 carotenoids are unique to the archaeal world. α-carotene was reported by some authors in Archaea [86], but its presence in prokaryotes is doubtful due to the trace amounts found and insufficient analysis.

Carotenoids or Apocarotenoids Present in the Three Domains of Life	Carotenoids Common to Archaea and Eukaryotes	Carotenoids Common to Archaea and Bacteria	Archaea-Specific Carotenoids
β-Carotene	(13*Z*)-β-Carotene (can also be found in bacteria from the photoisomerization of (all-*E*) -β-Carotene).	Bacterioruberin (BR)	Dihydrobisanhydrobacterioruberin
			Dihydroisopentenyldehydrorhodopin
Lycopene or (all-*E*)-Lycopene			3′,4′-Dihydromonoanhydrobacterioruberin
		Bisanhydrobacterioruberin (BABR)	1′,2′-Epoxy-2′-(2,3-epoxy-3-methylbutyl)-2-(3-hydroxy-3-methylbutyl)-3′,4′-didehydro-1,2,1′,2′-tetrahydro-ψ,ψ-caroten-1-ol
Phytoene or (15*Z*)-Phytoene			2-Isopentenyl-3,4-dehydrorhodopin
			3,4,3′,4′-Tetrahydrobisanhydrobacterioruberin
Phytofluene or (15*Z*)-Phytofluene			Trisanhydrobacterioruberin
		Monoanhydrobacterioruberin (MABR)	(9Z)-Zeaxanthin-3′-rhamnoside
			(13Z)-Zeaxanthin-3′-rhamnoside
			(15Z)-Zeaxanthin-3′-rhamnoside
(all-*E*)-Phytofluene		Zeaxanthin diglucoside	Zeaxanthin dirhamnoside
			(9Z)-Zeaxanthin dirhamnoside
Retinal or Vitamin A aldehyde (apocarotenoid)			Zeaxanthin monorhamnoside

**Table 2:** Archaea carotenoids and apocarotenoids classified according to their number of carbon atoms and chemical structure. For each molecule, the names of the producing Archaea species identified so far are presented.

Carotenoid Common Name	Chemical Name and Raw Formula	Structure	Archaea-Producing Species
**C_40_ Hydrocarbons**			
Astaxanthin	3,3′-Dihydroxy-β,β-carotene-4,4′-dione C_40_H_52_O_4_	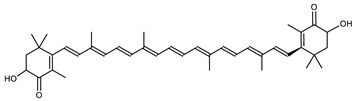	*Haloferax alexandrinus*–Archaea: Euryarchaeota [4]
β-Carotene	β,β-carotene C_40_H_56_	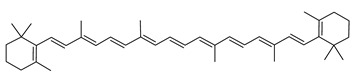	*Haloarcula japonica*–Archaea: Euryarchaeota [87] *Halobacterium cutirubrum*–Archaea: Euryarchaeota [86] *Halorubrum chaoviator Halo-G*–Archaea: Euryarchaeota [88]
(13*Z*)-β-Carotene	(13*Z*)-β,β-carotene C_40_H_56_	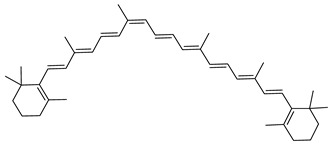	*Halobacterium cutirubrum*–Archaea: Euryarchaeota [86]
Canthaxanthin	β,β-Carotene-4,4′-dione C_40_H_52_O_2_	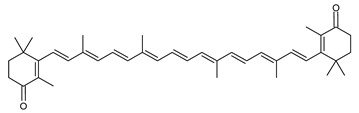	*Haloferax alexandrinus*–Archaea: Euryarchaeota [4]
Lycopene or (all-*E*)-Lycopene	ψ,ψ-carotene C_40_H_56_	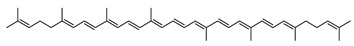	*Haloarcula japonica*–Archaea: Euryarchaeota [87] *Haloferax alexandrinus GUSF-1*–Archaea: Euryarchaeota [4] *Halorubrum chaoviator Halo-G*–Archaea: Euryarchaeota [88] *Haloterrigena turkmenica*–Archaea: Euryarchaeota [89]
Phytoene or (15*Z*)-Phytoene	(15*Z*)-7,8,11,12,7′,8′,11′,12′-octahydro-ψ,ψ-carotene C_40_H_64_	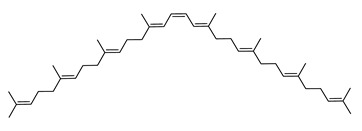	*Halobacterium cutirubrum*–Archaea: Euryarchaeota [86] *Haloferax alexandrinus GUSF-1*–Archaea: Euryarchaeota [4] *Haloterrigena turkmenica*–Archaea: Euryarchaeota [89]
Phytofluene or (15*Z*)-Phytofluene	(15*Z*)-7,8,11,12,7′,8′-hexahydro-ψ,ψ-carotene C_40_H_62_	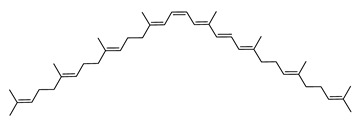	*Halobacterium cutirubrum*–Archaea: Euryarchaeota [86] *Haloferax alexandrinus GUSF-1*–Archaea: Euryarchaeota [4] *Haloterrigena turkmenica*–Archaea: Euryarchaeota [89]
(all-*E*)-Phytofluene	7,8,11,12,7′,8′-hexahydro-ψ,ψ-carotene C_40_H_62_	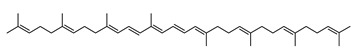	*Halobacterium cutirubrum*–Archaea: Euryarchaeota [86]
Zeaxanthin diglucoside	(3R,3′R)-3,3′-di(β-d-glucopyranosyloxy)-β,β-carotene C_52_H_76_O_10_	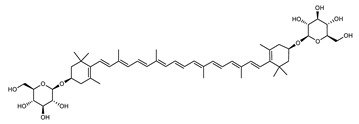	*Sulfolobus shibatae*–Archaea: Crenarchaeota [90]
(9*Z*)-Zeaxanthin-3′-Rhamnoside	(9*Z*,3R,3′R)-3′-(α-l-rhamnopyranosyloxy)-β,β-caroten-3-ol C_46_H_66_O_6_	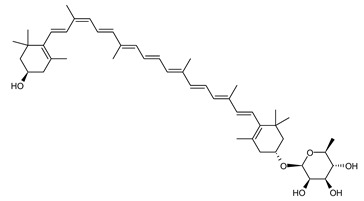	*Sulfolobus shibatae*–Archaea: Crenarchaeota [90]
(13*Z*)-Zeaxanthin-3′-Rhamnoside	(13*Z*,3R,3′R)-3′-(α-l-rhamnopyranosyloxy)-β,β-caroten-3-ol C_46_H_66_O_6_	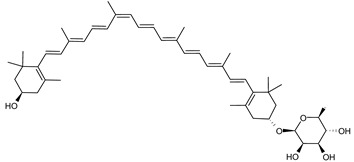	*Sulfolobus shibatae*–Archaea: Crenarchaeota [90]
(15*Z*)-Zeaxanthin-3′-Rhamnoside	(15*Z*,3R,3′R)-3′-(α-l-rhamnopyranosyloxy)-β,β-caroten-3-ol C_46_H_66_O_6_	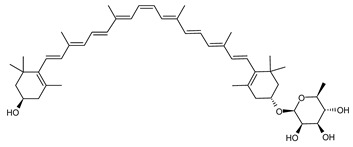	*Sulfolobus shibatae*–Archaea: Crenarchaeota [90]
Zeaxanthin dirhamnoside	(3R,3′R)-3,3′-di-(α-l-rhamnopyranosyloxy)-β,β-carotene C_52_H_76_O_10_	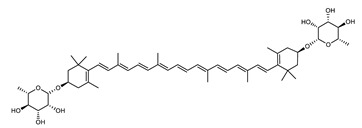	*Sulfolobus shibatae*–Archaea: Crenarchaeota [90]
(9*Z*)-Zeaxanthin dirhamnoside	(9*Z*,3R,3′R)-3,3′-di-(α-l-rhamnopyranosyloxy)-β,β-carotene C_52_H_76_O_10_	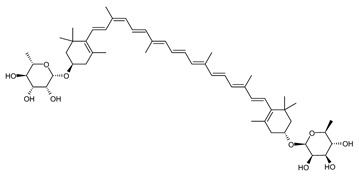	*Sulfolobus shibatae*–Archaea: Crenarchaeota [90]
Zeaxanthin monorhamnoside	(3R,3′R)-3′-(α-l-rhamnopyranosyloxy)-β,β-caroten-3-ol C_46_H_66_O_6_	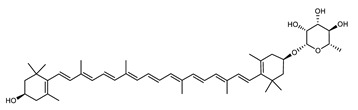	*Sulfolobus shibatae*–Archaea: Crenarchaeota [90]
**C_45_ Hydroxycarotenoids**			
Dihydroisopentenyldehydrorhodopin	(2S)-2-(3-methylbut-2-enyl)-1,2-dihydro-ψ,ψ-caroten-1-ol C_45_H_66_O	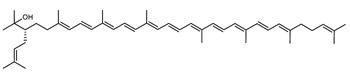	*Haloarcula japonica*–Archaea: Euryarchaeota [87]
2-Isopentenyl-3,4-dehydrorhodopin	(2S)-2-(3-methylbut-2-enyl)-3,4-didehydro-1,2-dihydro-ψ,ψ-caroten-1-ol C_45_H_64_O	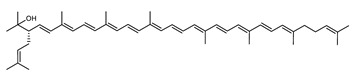	*Haloarcula japonica*–Archaea: Euryarchaeota [87]
**C_50_ Hydroxycarotenoids**			
Bacterioruberin (BR)	(2S,2′S)-2,2′-bis-(3-hydroxy-3-methylbutyl)-3,4,3′,4′-tetrahydro-1,2,1′,2′-tetrahydro-ψ,ψ-carotene-1,1′-diol C_50_H_76_O_4_	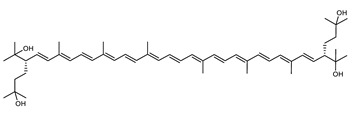	*Haloarcula japonica*–Archaea: Euryarchaeota [87] *Halobacterium salinarium*–Archaea: Euryarchaeota [61] *Halobacterium strain SP-2*–Archaea: Euryarchaeota [91] *Halococcus morrhuae*–Archaea: Euryarchaeota [61] *Haloferax alexandrinus GUSF-1*–Archaea: Euryarchaeota [4] *Halorubrum chaoviator Halo-G*–Archaea: Euryarchaeota [88] *Halorubrum strain SP-4*–Archaea: Euryarchaeota [91] *Haloterrigena turkmenica*–Archaea: Euryarchaeota [89] also present in *Micrococcus roseus* [92]
Bisanhydrobacterioruberin (BABR)	(2S,2′S)-2,2′-bis-(3-methylbut-2-enyl)-3,4,3′,4′-tetradehydro-1,2,1′,2′-tetrahydro-ψ,ψ-carotene-1,1′-diol C_50_H_72_O_2_	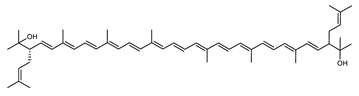	*Haloarcula japonica*–Archaea: Euryarchaeota [87] *Halobacterium salinarium*–Archaea: Euryarchaeota [61] *Halococcus morrhuae*–Archaea: Euryarchaeota [61] *Haloferax alexandrinus GUSF-1*–Archaea: Euryarchaeota [4] *Haloterrigena turkmenica*–Archaea: Euryarchaeota [89] also present in *Micrococcus roseus* [92] and *Arthrobacter glacialis* [93]
Dihydrobisanhydrobacterioruberin (DH-BABR)	(2S,2′S)-2,2′-bis-(3-methylbut-2-enyl)-3,4-didehydro-1,2,1′,2′-tetrahydro-ψ,ψ-carotene-1,1′-diol C_50_H_74_O_2_	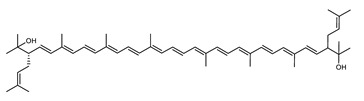	*Haloarcula japonica*–Archaea: Euryarchaeota [87]
3′,4′-Dihydromonoanhydrobacterioruberin	(2S,2′R)-2-(3-hydroxy-3-methylbutyl)-2′-(3-methylbut-2-enyl)-3,4-didehydro-1,2,1′,2′-tetrahydro-ψ,ψ-carotene-1,1′-diol C_50_H_76_O_3_	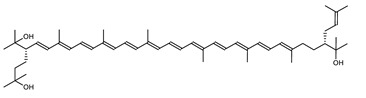	*Haloarcula japonica*–Archaea: Euryarchaeota [87]
1′,2′-Epoxy-2′-(2,3-epoxy-3-methylbutyl)-2-(3-hydroxy-3-methylbutyl)-3′,4′-didehydro-1,2,1′,2′-tetrahydro-ψ,ψ-caroten-1-ol	1′,2′-epoxy-2′-(2,3-epoxy-3-methylbutyl)-2-(3-hydroxy-3-methylbutyl)-3′,4′-didehydro-1,2,1′,2′-tetrahydro-ψ,ψ-caroten-1-ol C_50_H_74_O_4_	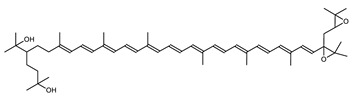	*Halobacterium* sp.–Archaea: Euryarchaeota
Haloxanthin	(2R,2′R)-2′-(3-Methylbut-2-enyl)-2-(3-methyl-1,3-peroxybutyl)-3,4-didehydro-1,2,1′,2′-tetrahydro-ψ,ψ-carotene-1,1′-diol C_50_H_74_O_4_	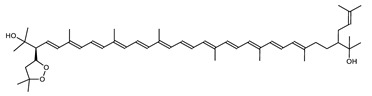	*Haloferax alexandrinus-* Archaea: Euryarchaeota [4]
Monoanhydrobacterioruberin (MABR)	2-(3-hydroxy-3-methylbutyl)-2′-(3-methylbut-2-enyl)-3,4,3′,4′-tetradehydro-1,2,1′,2′-tetrahydro-ψ,ψ-carotene-1,1′-diol C_50_H_74_O_3_	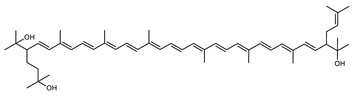	*Haloarcula japonica*–Archaea: Euryarchaeota [87] *Halobacterium* sp.–Archaea: Euryarchaeota *Haloferax alexandrinus GUSF-1*–Archaea: Euryarchaeota [4] *Haloterrigena turkmenica*–Archaea: Euryarchaeota [89] also present in *Micrococcus roseus* [92]
3,4,3′,4′-Tetrahydrobisanhydrobacterioruberin	(2R,2′R)-bis-(3-methylbut-2-enyl)-1,2,1′,2′-tetrahydro-ψ,ψ-carotene-1,1′-diol C_50_H_76_O_2_	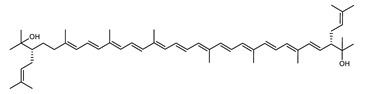	*Haloarcula japonica*–Archaea: Euryarchaeota [87]
Trisanhydrobacterioruberin	2,2′-bis(3-methylbut-2-enyl)-3,4,3′,4′-tetradehydro-1,2-dihydro-ψ, ψ-caroten-1-ol C_50_H_70_O	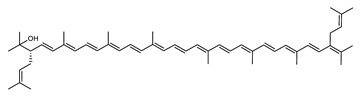	*Halobacterium salinarium*–Archaea: Euryarchaeota [61] *Halobacterium* sp.–Archaea: Euryarchaeota *Halococcus morrhuae*–Archaea: Euryarchaeota [61]
**Apocarotenoids**			
Retinal or Vitamin A aldehyde	15-apo-β-caroten-15-al C_20_H_28_O	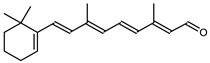	*Haloarcula japonica*–Archaea: Euryarchaeota [87] *Halobacterium salinarum*–Archaea: Euryarchaeota [94]

**Table 3:** Carotenoids composition of Archaea ecophysiological groups. For each group, the carotenoids composition is detailed, when available in the literature. The carotenoid composition of some genera such as *Pyrodictium* or ecophysiological groups such as methanogens and psychrophiles have not been described yet.

Archaea Classification	Carotenoid Composition	Species or Genus Studied
Thermophiles and hyperthermophiles	β-Carotene Lycopene or (all-*E*)-Lycopene Phytoene or (15*Z*)-Phytoene (9*Z*)-Zeaxanthin-3′-Rhamnoside (13*Z*)-Zeaxanthin-3′-Rhamnoside (15*Z*)-Zeaxanthin-3′-Rhamnoside Zeaxanthin diglucoside (9*Z*)-Zeaxanthin dirhamnoside Zeaxanthin dirhamnoside Zeaxanthin monorhamnoside	*Sulfolobus shibatae*–Archaea: Crenarchaeota [90]
Halophiles	Astaxanthin Bacterioruberin (BR) β-Carotene (13*Z*)-β-Carotene Bisanhydrobacterioruberin (BABR) Canthaxanthin (all-*E*)-Phytofluene Dihydrobisanhydrobacterioruberin (DH-BABR) Dihydrobisanhydrobacterioruberin Dihydroisopentenyldehydrorhodopin 3′,4′-Dihydromonoanhydrobacterioruberin 1′,2′-Epoxy-2′-(2,3-epoxy-3-methylbutyl)-2-(3-hydroxy-3-methylbutyl)-3′,4′-didehydro-1,2,1′,2′-tetrahydro-ψ,ψ-caroten-1-ol Haloxanthin 3-Hydroxy-echinenone 2-Isopentenyl-3,4-dehydrorhodopin Lycopene or (all-*E*)-Lycopene Monoanhydrobacterioruberin (MABR) Phytoene or (15*Z*)-Phytoene Phytofluene or (15*Z*)-Phytofluene Retinal (apocarotenoid) 3,4,3′,4′-Tetrahydrobisanhydrobacterioruberin Trisanhydrobacterioruberin	*Halobacterium cutirubrum*–Archaea: Euryarchaeota [86] *Haloarcula japonica*–Archaea: Euryarchaeota [45,87] *Haloferax alexandrinus*–Archaea: Euryarchaeota [4] *Halorubrum chaoviator Halo-G*–Archaea: Euryarchaeota [88] *Halococcus morrhuae*–Archaea: Euryarchaeota [86] *Haloferax alexandrinus GUSF-1*–Archaea: Euryarchaeota [4] *Haloterrigena turkmenica*–Archaea: Euryarchaeota [89] Described in *Haloferax volcanii* [76,95,96]
Methanogens	Unknown	Methanogens are unstudied for their carotenoid and apocarotenoids composition. The group contains various genera such as *Methanopyrus* (both methanogenic and thermophilic), *Methanosarcina, Methanomicrococcus and Methanosaeta*
Psychrophiles	Unknown	Psychrophiles are unstudied for their carotenoid and apocarotenoids composition. Some examples of psychrophilic species are *Methanococcoides burtonii* and *Methanogenium frigidum*

**Table 4:** Enzymes and related genes involved in the biosynthesis of Archaea-specific carotenoids. Numbers in parenthesis indicate the enzyme number or EC number.

Carotenogenesis Pathway after Phytoene Formation	Gene	Enzyme
Bacterioruberin biosynthetic pathway	CrtD (1.3.99.37) CruF (4.2.1.161) LyeJ (2.5.1.1.50)	3,4-desaturase 2”,3”-hydratase Bifunctional lycopene elongase and 1,2-hydratase
Lycopene biosynthetic pathway	CrtB (2.5.1.32) CrtE (2.5.1.29) CrtI (K10027) FDPS (2.5.1.10)	15-cis-phytoene synthase Geranylgeranyl diphosphate synthase Phytoene desaturase Farnesyl diphosphate synthase
Retinal biosynthetic pathway	Brp (1.13.11.63) CrtYcd (5.5.1.19)	β-carotene dioxygenase Lycopene β-cyclase
Zeaxanthin biosynthetic pathway	CrtYcd (5.5.1.19) CrtZ (1.14.15.24)	Lycopene β-cyclase β-carotene 3-hydroxylase

**Table 5:** Review of Archaea carotenoids biological and pharmacological activities according to the literature.

Common Name	Biological Activities and Properties
**C_40_ Hydrocarbons**	
β-Carotene	Photosynthetic pigment present in all organisms making oxygenic photosynthesis from cyanobacteria to higher plants [108,109] Photoprotective agent [110] Present in the reaction-center complexes (RC) and the light-harvesting complexes (LHC) of photosystem I (PSI) as well as the RC and the core LHC of photosystem II (PSII) [111,112,113] Provitamin A [114,115] Antioxidant—free radical scavenger/singlet oxygen quencher, 101 times stronger than that of α-tocopherol (SOAC value: 101) [110,111,116,117,118,119] Anti-apoptotic agent (mouse model of traumatic brain injury) preventing loss of Bcl2, preventing accumulation of Bax, and preventing accumulation or activation of Caspase 3 [120] β-carotene-derived retinoid acids bind with retinoid acid receptor (PAR) and retinoid X receptor. Receptors dimerization leads to a functional transcription factor regulating gene expression during neurogenesis. Neuroprotective activity against apoptosis [120] Anticarcinogenic activity [121,122] Cell differentiation and proliferation promoter by upregulating Connexin 43 gene [123] Immune response enhancement in animals and humans [124] Found in human skin throughout the epidermis, dermis and also the subcutaneous [125]
(13*Z*)-β-Carotene	Provitamin A activity (10% of that of all-trans-β-carotene) [126,127]
Lycopene or (all-*E*)-Lycopene	Photoprotection [114] Radioprotection against gamma-radiation-induced cellular damages [128] Strong antioxidant —strong singlet-quenching ability—141 times stronger than that of α-tocopherol (SOAC value: 141) [111,114,116,117,118,119] Protecting mitochondria and mitochondrial DNA by antioxidant properties and treatment with lycopene prevents loss of mitochondrial inner membrane potential during ROS challenge [120] Antiradical activity [129] Found in human skin throughout the epidermis, dermis and also the subcutaneous [125] Anticarcinogenic activity by reducing insulin growth factor 1 (IGF-1) stimulation with an increase in membrane-associated IGF-binding proteins; also slows down IGF-1-stimulated cell cycle progression [114,116,121,122,130] Inhibits the proliferation of androgen-dependent human prostate tumor cells through activation of PPARγ-LXRα-ABCA1 [131] Anti-apoptotic agent—preventing loss of Bcl2 and Bcl-xL, preventing accumulation of Bax, preventing accumulation or release of Cytochrome C, and preventing accumulation or activation of Caspase 3 [120] Anti-inflammation [114] Anti-inflammatory effects of lycopene may help alleviate neuropsychiatric diseases such as post-traumatic stress disorder and depression [120] Antimicrobial activity against *S. aureus*, and *E. coli* O-157 [132] Antifungal activity against *C. albicans* by arresting their cell cycle [132] Cell differentiation and proliferation promoter by upregulating Connexin 43 gene [123] Non-Provitamin A [114]
Phytoene	Anticarcinogenic activity [121]
Phytofluene	Anticarcinogenic activity—more active than β-carotene [122]
(all-*E*)-Phytofluene	No biological activity reported
Zeaxanthin diglucoside	No biological activity reported
(9*Z*)-Zeaxanthin-3′-rhamnoside	No biological activity reported
(13*Z*)-Zeaxanthin-3′-rhamnoside	No biological activity reported
(15*Z*)-Zeaxanthin-3′-rhamnoside	No biological activity reported
Zeaxanthin dirhamnoside	No biological activity reported
(9*Z*)-Zeaxanthin dirhamnoside	No biological activity reported
Zeaxanthin monorhamnoside	No biological activity reported
**C_45_ Hydroxycarotenoids**	
Dihydroisopentenyldehydrorhodopin (DH-IDR)	No biological activity reported
2-Isopentenyl-3,4-dehydrorhodopin (IPR)	No biological activity reported
**C_50_ Hydroxycarotenoids**	
Bacterioruberin (BR)	Antioxidant activity—much better radical scavenger than that of β-carotene as it contains 13 pairs of conjugated double bonds [45,133] limits oxidation due to H_2_O_2_ exposure [80,134] Photoprotective activity—limits oxidative DNA damage from UV irradiation [80,134] Radio protective activity—limits oxidative DNA damage from gamma irradiation [80,134]
Bisanhydrobacterioruberin (BABR)	No biological activity reported
Dihydrobisanhydrobacterioruberin (DH-BABR)	No biological activity reported
3′,4′-dihydromonoanhydrobacterioruberin	No biological activity reported
1′,2′-epoxy-2′-(2,3-epoxy-3-methylbutyl)-2-(3-hydroxy-3-methylbutyl)-3′,4′-didehydro-1,2,1′,2′-tetrahydro-ψ,ψ-caroten-1-ol	No biological activity reported
Monoanhydrobacterioruberin (MABR)	No biological activity reported
3,4,3′,4′-tetrahydrobisanhydrobacterioruberin	No biological activity reported
Trisanhydrobacterioruberin	No biological activity reported
**Apocarotenoids**	
Retinal or Vitamin A aldehyde	Photoreception in human retina Abolishes the function of the toxin suberitine at a stoichiometric ratio of 1:1 [135] Isomerized to 13*Z*-retinal under light, and isomerized back to all-trans retinal in the dark
